# Upper bill bending as an adaptation for nectar feeding in hummingbirds

**DOI:** 10.1098/rsif.2024.0286

**Published:** 2024-11-27

**Authors:** Alejandro Rico-Guevara, Diego Sustaita, Kristiina J. Hurme, Jenny E. Hanna, Sunghwan Jung, Daniel J. Field

**Affiliations:** ^1^ Department of Biology, University of Washington, Seattle, WA 98195, USA; ^2^ Burke Museum of Natural History and Culture, University of Washington, Seattle, WA 98195, USA; ^3^ Department of Biological Sciences, California State University San Marcos, 333 S. Twin Oaks Valley Rd, San Marcos, CA 92096, USA; ^4^ Independent Researcher, Babraham, Cambridgeshire CB22 3AG, UK; ^5^ Department of Biological and Environmental Engineering, Cornell University, Ithaca, NY 14850, USA; ^6^ Department of Earth Sciences, University of Cambridge, Cambridge CB2 3EQ, UK; ^7^ Museum of Zoology, University of Cambridge, Cambridge CB2 3EJ, UK

**Keywords:** amphikinesis, geometric morphometrics, nectarivory, rhynchokinesis, flexural rigidity

## Abstract

Observations of maxillary (upper bill) bending in hummingbirds have been considered an optical illusion, yet a recent description of out-of-phase opening and closing between their bill base and tip suggests a genuine capacity for bill bending. We investigate bill kinematics during nectar feeding in six species of hummingbirds. We employed geometric morphometrics to identify bending zones and combined these data with measurements of bill flexural rigidity from micro-computed tomography scans to better understand the flexing mechanism. We found that the mandible remains in place throughout the licking cycle, while the maxilla undergoes significant shape deformation, such that the distal portion of the upper bill bends upwards. We propose that bill bending is a key component of the drinking mechanism in hummingbirds, allowing the coordination of bill function (distal wringing and basal expansion) and tongue function (raking/squeegeeing) during intra-oral transport. We present a fluid analysis that reveals a combination of pressure-driven (Poiseuille) and boundary-driven (Couette) flows, which have previously been thought to represent alternative drinking mechanisms. Bill bending allows for separation of the bill tips while maintaining a tightly closed middle section of the bill, enabling nectar exploitation in long and narrow flowers that can exclude less efficient pollinators.

## 1. Introduction

Despite often exhibiting lightweight, pneumatized skeletons, the bones of birds are usually extremely rigid [1]. Bone flexibility is generally limited in most tetrapods [2]; however, some groups of birds exhibit flexible bones in their skulls for functional reasons (e.g. [3–5]). The bill is one of the most evolutionarily labile external structures in birds, reflecting the immense disparity of feeding styles exhibited across roughly 11 000 extant species. In addition to this remarkable ecomorphological disparity, the striking dexterity of the avian bill is enhanced by variable degrees of cranial kinesis, the origin of which may have helped compensate for the loss of manual dexterity associated with the origin of avian flight [6], and at least an incipient degree of cranial kinesis appears to have been present in the last common ancestor of all extant birds [7]. The most common form of avian cranial kinesis (prokinesis [3]) involves movement of the entire upper bill with respect to the cranium by flexion of a region of narrow bone where the beak meets the cranium, known as the craniofacial or nasofrontal hinge [8–13]. However, some bird clades exhibit additional specializations for bill flexion in which the very bones of the bill are capable of bending, such as lateral bowing of the lower jaws (e.g. [4,14,15]) and dorsiflexion of the upper jaws (i.e. rhynchokinesis [16,17]).

Although all birds possess skulls that are kinetic to an extent [16], rhynchokinesis near the bill tip is especially well developed in shorebirds (Charadriiformes: Scolopacidae). This enables many shorebird species to open and close the tips of the bill independently of the bill base, which is generally associated with their substrate-probing feeding habits [16,18,19] but has also been shown to help facilitate the unique surface-tension transport mechanism of phalaropes [20,21] and enhance feeding efficiency on small aquatic prey in Calidris sandpipers [22,23]. However, bill bending is considerably less well understood outside of shorebirds, and few other avian clades are known to exhibit this specialization. An additional group capable of bill bending is Strisores, which in part includes nightjars (Caprimulgidae) and Apodiformes (e.g. swifts and hummingbirds) [24–26]. Most subclades within Strisores are aerial insectivores that employ mandibular bending for gape expansion [14], and the lower jaws of hummingbirds have been observed to bend ventrally and laterally during flycatching manoeuvres and visual displays, indicating considerable flexibility of their lower jaws [15,27,28]. Hummingbird ancestors were short-billed aerial predators resembling modern cypselomorphs such as swifts (Apodidae [29,30]). Through coevolution with increasingly specialized ornithophilous plants, hummingbird beaks became long and slender [30]. To be able to take advantage of nectar as a food resource without damaging the flower (allowing continued nectar production), hummingbird tongues became elongated and protrusible, evolving as highly efficient liquid-collecting devices closely coupled with, and paralleling, elongation and thinning of their bills to fit inside narrow flowers (reviewed by Rico-Guevara *et al*. [31]). Interestingly, such a high degree of morphofunctional specialization for nectarivory has not compromised their ability to effectively perform aerial insectivory (the plesiomorphic dietary state for Strisores), in large part because of mandibular bending. Yanega & Rubega [15] demonstrated that ventral flexion of the mandible (lower jaw) enhances aerial prey capture success. Here, we evaluate for the first time whether another form of bill bending—distal rhynchokinesis of the upper jaw—exists in hummingbirds and whether it confers advantages for enhancing drinking efficiency.

From a mechanical perspective, ‘drinking’ is simply an animal’s behaviour of transporting fluid into the body. There are two fundamental modes of fluid transport: pressure-driven (Poiseuille) and boundary-driven (Couette) flows [32–34]. Most animals employ a pressure-driven mechanism (suction) using a confined oral structure, while a few species (mostly carnivoran mammals) employ boundary-driven flow (lapping) [35–37]. For small animals, capillary-driven flows can be used, which can be categorized as pressure-driven flow to some extent [38–41]. Although widely hypothesized [42], capillary filling of the tongue during nectar feeding in birds has only been documented in pied honeyeaters [43], while other honeyeater species (Passeriformes: Meliphagidae) employ a similar mechanism to hummingbirds, the most intensively studied group of avian nectarivores (recent reviews in [42–44]).

Previous research on hummingbird drinking biomechanics has shown how the tongue collects nectar [45,46] and how this liquid is offloaded inside the bill [45]. Briefly, during tongue protrusion, hummingbirds flatten the distal grooves of their tongues [45,46], which are hollow to collect the nectar but comprise only approximately half of their tongue length [47], so they cannot be used as straws. While the tongue moves through the air during protrusion, the elastic energy loaded into the groove walls during the flattening is conserved by a remaining layer of liquid inside the grooves acting as an adhesive [45]. When the tongue touches the nectar, the fringed tip opens up [48], and the supply of fluid allows the release of the elastic energy, which expands the grooves and pulls the nectar to fill the entire tongue grooves [45]. The tongue is then retracted, the fringes at the tip capture fluid via a natural origami self-folding trap [48], and the tongue, now sealed at the tip and completely filled with nectar, is quickly brought inside the oral cavity while the bill tips are kept separated. As soon as the tongue is inside the bill, the bill tips close leaving only a small gap through which the tongue grooves are protruded, wringing the nectar inside the oral cavity near the bill tip, flattening the tongue and starting the process again with the next cycle [45], with a frequency of approximately 15 Hz [31]. In spite of these advances in our understanding of the nectar collection mechanisms, however, the process by which nectar is transported to the throat during hummingbird feeding has only recently been described [49], and the underlying mechanics of the bill motions that foster this intra-oral transport are unknown.

The default expectation for bill separation, set by a lever mechanism wherein the jaws hinge upon the nasofrontal and quadrato-mandibular joints at the bill base [5], is that separation at the base is followed by in-phase synchronized (same period) separation of the tip—the same way in which a pair of scissors works. Hummingbirds, on the contrary, exhibit phase-shifted (out of phase) separation between the base and the tip, with the bill’s middle portion showing the least amount of separation [49], akin to the motion of a seesaw. However, the mechanism by which this phase-shifted bill opening operates has yet to be explained. It could potentially be achieved by different mechanisms such as mandibular bending (lower bill tip deflecting downwards), mandibular rotation (a curved lower jaw elevated basally and hinging in the middle of the upper jaw), maxillary bending (upper bill tip deflecting upwards), or combinations of the above mechanisms. Nitzsch [50] first observed hinges in the still flexible bills of juvenile hummingbirds and inferred that hummingbirds could use bending of the tip of the maxilla to wring nectar off the tongue during extrusion. Such bill bending might optimize the feeding process through the coupling of bill and tongue movements (see [51–53]), and although this was never experimentally demonstrated, Bühler [14] also suggested potential flexion zones along the maxilla.

Despite these early studies hypothesizing a role for bill bending during hummingbird feeding, Zusi’s [16] exhaustive comparative survey of avian rhynchokinesis (i.e. bending of the upper bill) found no osteological evidence supporting distal bill bending in hummingbirds. Zusi [16], therefore, concluded that most species of hummingbirds were proximally rhynchokinetic, and a few actually prokinetic, meaning that any dorsally directed bending of the upper jaw would be restricted to the base of the bill. However, Zusi [17] later acknowledged the slight bending of the tip of the upper jaw of hummingbirds observed in videos by Rico-Guevara & Rubega [48] and concluded that distal rhynchokinesis was not only possible but could even facilitate nectar consumption [17]. Furthermore, Zusi [17] underscored the need for ‘precise measurements of cranial kinesis of live hummingbirds and knowledge of the physical properties of the structural components of the prepalatal upper jaw’.

Here, we tested the hummingbird bill-bending hypothesis by combining high-speed videography, geometric morphometrics and micro-computed tomography (µCT) to quantify bill bending *in vivo* and *ex vivo* across a wide phylogenetic sample of hummingbirds (Strisores: Trochilidae). Our objective was to quantify the upper bill flexibility of hummingbirds during nectar feeding and to determine whether the hummingbird’s upper jaws exhibit a distinct dorsoventral flexural rigidity profile that facilitates bending of the distal portion of the bill. These insights help clarify the specialized form of nectar drinking exhibited by approximately 350 species of extant hummingbirds.

## 2. Methods

### 2.1. Video analyses

We filmed free-living hummingbirds trained to drink nectar from artificial feeders with clear sides, which were wide enough to avoid limiting any bill tip motion during feeding (e.g. [46,48]), in Colombia, Ecuador and the United States (all at private locations with landowner permission). We worked with adult birds (judging by the absence of many visible corrugations at the bill base [54–56]) drinking while hovering (e.g. electronic supplementary material, video S1), with high-speed cameras (TroubleShooter HR, Fastec Imaging, 1000 frames s^−1^, 1280 × 512 pixels) coupled with macro lenses (Nikon 105 mm f/2.8 VR). All filming activities were reviewed and authorized by the Institutional Animal Care and Use Committee at the University of Washington (protocols 4498-01, 4498-03) and at the University of Connecticut (exemption number E09-010). We processed videos from representative subjects of six species of hummingbirds belonging to four of the nine major hummingbird subclades to maximize phylogenetic, morphological and functional diversity in our sample for assessing the phylogenetic distribution of bill bending in Trochilidae (electronic supplementary material, table S10). For each of the six subjects, high-speed videos of licking footage (e.g. electronic supplementary material, video S1) were converted into a series of image files using QuickTime Player v. 7.6.6. All images in each sequence were digitally and equally enhanced by consistently adjusting the contrast and brightness using ImageJ 1.45 p [57], to maximize the visibility of targeted landmarks (dorsal culmen curvature and tomium).

We measured 10 licking cycles (i.e. ‘licks’) per individual (e.g. electronic supplementary material, video S1), with the exception of *Chalybura buffonii*, for which only five cycles were analysed. We defined the start of a lick as the first frame in which the tongue is visible while being protracted, and the end of each lick as the last frame before the tongue is visible again during protraction. The duration of one lick was usually less than 0.1 s, and we extracted a subset of 11 equally spaced frames from each high-speed video sequence to analyse (yielding 10 time steps approx. 10 ms apart). For the analyses, the 1st (‘frame 0’) and 11th (‘frame 10’) frames were the ones directly preceding the tongue’s first appearance during protraction, thus encompassing a full cycle.

### 2.2. Assessment of bill deformation

**Figure 1.**
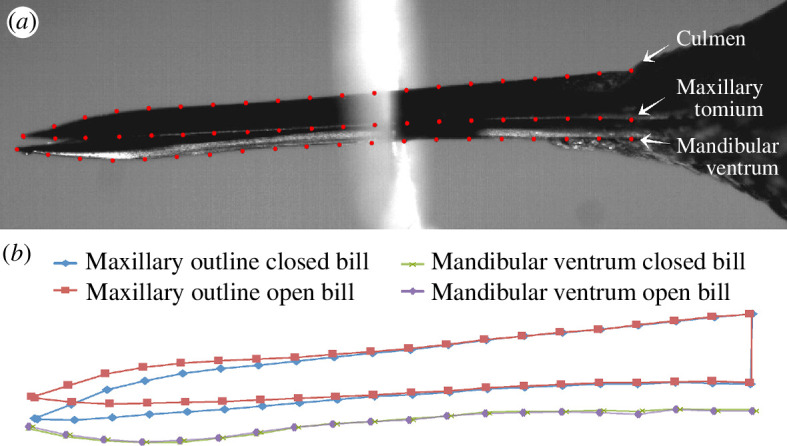
(*a*) Description of semi-landmarks used for video analyses. (*b*) Outputs from a geometric morphometric analysis of the landmarks showing the most extreme displacements of the upper bill (blue and red) and the visible bottom of the mandible (green and purple) during a given licking cycle.

Starting with the lateral views and for each of the 11 frames per lick, we digitally traced three bill contours to create profile lines using tpsDIG2 [58]. The first line followed the culmen beginning distally at the maxillary tip and ending at the most proximal point of the exposed culmen (the distal-most extent of feathering). A vertical line perpendicular to the bill axis was followed ventrally from the most proximal point of the exposed culmen, and its intercepts with the other bill contours were used to place points defining the most proximal points of the other profile lines: one on the maxillary tomium and one on the mandibular ventrum (figure 1*a*). We then traced the maxillary tomium profile from this proximal point (intercept with the end of exposed culmen perpendicular line) to the maxillary tip, as well as the profile of the mandibular ventrum (defined here as the lower jaw contour, that is, the exposed culmen’s counterpart on the lower jaw) from the most proximal point as defined above to the mandibular tip. These three bill profile lines were then resampled using tpsDIG2 so that each line had 21 equidistant semi-landmarks, with which we could assess bill deformation (figure 1*b*). Landmark tracking was ultimately performed by a few different observers, so we conducted a repeatability experiment (electronic supplementary material, S1) to assess the potential impacts of within- and among-observer tracking error, in a manner similar to that described by Moccetti *et al*. [59].

We used a geometric morphometric approach to visualize the relative displacements of landmarks throughout a given licking cycle, using approaches adapted from [60–62]. This analysis allowed us to assess the deformation of the culmen (underlain by the premaxillary, nasal, maxillary and jugal bones) during the course of a lick, to help identify the bill’s bending zones. We used tpsRelw32 [63] to perform generalized Procrustes alignment (GPA; using generalized least squares) of all the frame image landmark configurations (‘frames’, from here on) of all lick sequences (collectively) for each subject filmed. We then performed a relative warps analysis (i.e. principal component analysis of the partial warp scores [63]) to validate the observed culmen deformations. For this analysis, we chose to slide semi-landmarks (by resampling along the contour lines) using the method that minimizes the Procrustes distance (d2), both based on recommendations of [64], as well as our own observations that a minimum bending energy approach seemed to introduce artefacts. We used Procrustes ANOVA, a non-parametric approach that leverages residual randomization permutation procedures, as described by Adams & Collyer [65,66] and Collyer & Adams [67], implemented in function procD.lm of the R package geomorph [68]. This procedure tests for the effects of independent variables on the full set of dependent (shape) variables (i.e. the Procrustes coordinates: the landmark coordinates aligned by generalized Procrustes analyses). We performed Procrustes ANOVAs for each subject to test the significance of the changes in bill shape observed across sequential frames. For these analyses, the effects of ‘dataset’ (in the intra- and inter-observer landmark repeatability analysis; electronic supplementary material, file S1) and ‘cycle’ (in the omnibus test of bill deformation for each species) were included as blocking factors. One advantage of this approach is its robustness to the unfavourably high p : n ratios inherent to geometric morphometric datasets [67]. Generalized least-squares analyses were performed using type III sums of squares and cross-products and were set to 9999 permutations of null model residuals. Wireframe plots for selected sequential frames were generated using the plotRefToTarget function of R package geomorph [68], with magnification set to 1.5–7× to better visualize the shape changes.

We summarized the total GPA variances (S^2^) for each landmark along the dorsal culmen and tomium. We felt that using the GPA variance (which provides a measure of the variability of the *x* and *y* positions of each Procrustes-aligned landmark) was appropriate and sufficient to fulfil our objectives of: (i) demonstrating upper bill movement at each landmark during a licking cycle and (ii) generating a quantitative variable for the relative displacements of individual landmarks along the length of the culmen over time to juxtapose with the flexural rigidity data described below. We acknowledge the potential shortcoming of this approach, in that during the Procrustes alignment procedure, the variance in landmark positions is distributed across all landmarks [69]. However, our preliminary investigation of this effect (by randomly varying pixel positions at some landmarks of a single frame within the ranges of *x* and *y* displacements observed during a lick cycle), indicated that the variance attributed to other (unvaried) landmarks is negligible, amounting to less than or equal to 5.7% (depending on the number of landmarks varied) of the variance in altered landmark positions and less than 1% of the total landmark variances observed during an actual lick cycle. We chose not to slide the semi-landmarks for this particular analysis, primarily because we wanted to observe the actual variation in landmark positions over the course of a lick cycle while avoiding any potential influence of statistical artefacts, as the sliding procedure tends to exaggerate variation in the vertical dimension (e.g. [64]), which is the primary locus of the signal that we endeavoured to detect.

### 2.3. High-resolution X-ray computed tomography

We generated µCT scans (down to 5 μm voxel resolution) of hummingbird heads from specimens from the Yale Peabody Museum corresponding to the same species studied, to discern between bone and rhamphotheca and thus match our high-speed videos to the bone density analyses (see §2.4 below). The specimens were scanned at The University of Texas High-Resolution X-ray Computed Tomography Facility, Austin, Texas, and on a µCT35 instrument (Scanco Medical AG) at the Yale Core Centre for Musculoskeletal Disorders, New Haven, Connecticut. Scans were performed at 70 kV and 10 W, with Xradia 0.5 and 4× objectives, and 1 mm SiO_2_, or no filter. Specimens were scanned in three parts, with the scans stitched together using Xradia plugins, and the voxel size was between 15.5 and 5.2 μm. The 16 bit TIFF images were reconstructed in Xradia Reconstructor, and the total number of slices per specimen was between 2223 and 2854, with scan times between 4 and 7 h.

### 2.4. Dorsoventral flexural rigidity analysis

A long-standing problem in vertebrate functional morphology has been the difficulty associated with quantifying the flexural rigidity profile of bones across their entire length. Although subjecting bones to mechanical tests to determine bending resistance provides a quantitative estimate of flexural rigidity at certain points along a bone, such tests provide little information about a bone’s overall mechanical design. A method of non-destructive flexural rigidity determination, BendCT, facilitates the quantification of a bone’s flexural rigidity profile along a user-defined bending axis across its entire length [4]. We used BendCT to test the hypothesis that the ‘bending zone’ of the hummingbird’s upper jaw is characterized by a marked decrease in the bone’s dorsoventral flexural rigidity, relative to proximal and distal rigid zones.

Flexural rigidity is the product of an object’s elastic stiffness, quantified as the Young’s modulus (*E*), and its cross-sectional shape, quantified as the second moment of area (*I*),


(2.1) $$Flexural\;Rigidity\;=E(I)=E\int_{A}y^{2}\;(dA),$$

where y is the distance between the area increment d*A* and the bending axis of interest. In bone, mineral density is the main determinant of *E*, allowing *E* to be estimated using equations relating experimentally derived values of *E* to non-invasive proxies of bone mineral density (e.g. Hounsfield units). The calibration of a Hounsfield unit–mineral density relationship was accomplished by scanning hydroxyapatite phantoms of known mineral density under the same conditions as the hummingbird specimens. We then followed previous work (e.g. [2,4,70]) by applying the regression derived by Ciarelli *et al*. [71] to estimate *E* averaged across all three orthogonal directions from mineral density. This approach ignores the potential influence of bone water content and material anisotropy on *E*. However, *EI*, using average values, does not take into account non-homogeneity in cross-sectional mineral density, which leads to regional variation in *E*. To address this issue, BendCT computes cross-sectional flexural rigidity pixel-by-pixel for each serial cross section, by defining d*A* as the area of a pixel, and taking the summation form of the integral as follows:


(2.2) $$\sum_{i=1}^{n}E(y_{i}^{2})\;dA_{i},$$

where *i* identifies an individual pixel and *n* is the total number of pixels in a user-defined region of interest. Thus, BendCT facilitates the quantification of flexural rigidity weighted by the effect of bone density on *E*, relative to the dorsoventral bending axis of the hummingbird’s upper jaw. Stacks of high-resolution µCT images were obtained for the upper jaws of the hummingbird species for which performance data were collected, from the nasofrontal hinge to the rostral tip of the bill. BendCT was used to reconstruct the pattern of dorsoventral flexural rigidity variation throughout the hummingbird’s upper jaw, to test the hypothesis that the hummingbird’s upper jaw bending is facilitated by depressed dorsoventral flexural rigidity within the jaw’s bending zone. This hypothesis predicts that regions of relatively high dorsoventral flexural rigidity should flank the bending zone at the bill’s distal and proximal ends; however, the bending zone itself should be characterized by a pronounced reduction in dorsoventral flexural rigidity. To examine this, we paired the dorsoventral flexural rigidity data (based on the combined frontal process of the premaxillae, defined as ‘top’, and the main body of the premaxillae, defined as the ‘fused’ portions of the bill only—see [17] for a discussion on the anatomical nomenclature of the hummingbird bill bones—to account for resistance to bending in the dorsoventral axis) with the kinematic geometric morphometrics data. Because the subjects filmed and the specimens µCT scanned were not the same individuals, we aligned the BendCT flexural rigidity data with the kinematic data on their own sets of x and y axes to assess the congruence between datasets. We scaled the lengths of the bills of the specimens from which the dorsoventral flexural rigidity data were obtained to the total culmen lengths estimated for each subject (i.e. from the rostral bill tip to the nasofrontal hinge), based on their measured exposed culmen lengths (i.e. from the rostral bill tip to the cranial extent of the rhamphotheca) and the relationship between exposed culmen and total culmen lengths measured from other specimens of the same species. This alignment of the datasets takes into account that the BendCT data distally-to-proximally start on the bony part of the bill, while the geometric morphometrics data start at the keratinous rostral tip of the culmen. We then used the CT scans themselves to measure the portion of the bill tip that is only keratin, resulting in a more accurate alignment.

## 3. Results

### 3.1. Rhynchokinetic bill deformation in live hummingbird subjects

**Figure 2.**
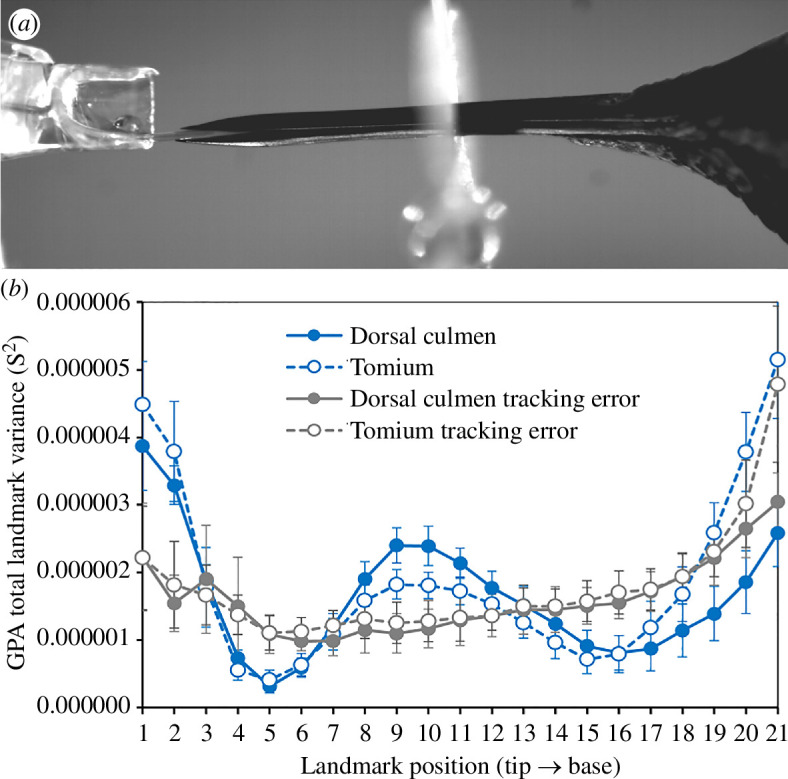
(*a*) Image from a *Calypte anna* lick cycle. (*b*) Mean ± 95% CI (*n* = 10 lick cycles) GPA total landmark variances (S^2^) computed over 11 sequential frames, for the dorsal culmen (blue filled symbols and solid line) and tomium (blue open symbols and dashed lines) of the *Calypte anna* subject. The mean ± 95% CI (*n* = 11 frames) GPA landmark variances due to tracking error obtained from a single lick cycle tracked by five different participants three times each (electronic supplementary material, file S1.1) are shown in grey (dorsal culmen filled symbols and solid line; tomium open symbols and dashed line).

The GPA landmark variances during lick cycles exceeded frame error variances (based on non-overlapping 95% CIs; electronic supplementary material, file S1.1) along landmark positions 1−2, 8−12 and 14−18 in *Calypte anna* (figure 2), indicating zones of mobility, or ‘bending zones’, where landmark variances were greater than tracking error (e.g. landmark positions 1–2 and 8–12; figure 2) punctuated by regions of no discernible mobility where variances were actually lower than error (e.g. landmark positions 4–6 and 14–18; figure 2). Based on the (conservative) error analyses of this subject, we are confident that the regions of high and low GPA variances in other subjects accurately reflect the zones of greatest and least mobility (respectively) along the lengths of their bills.

**Figure 3.**
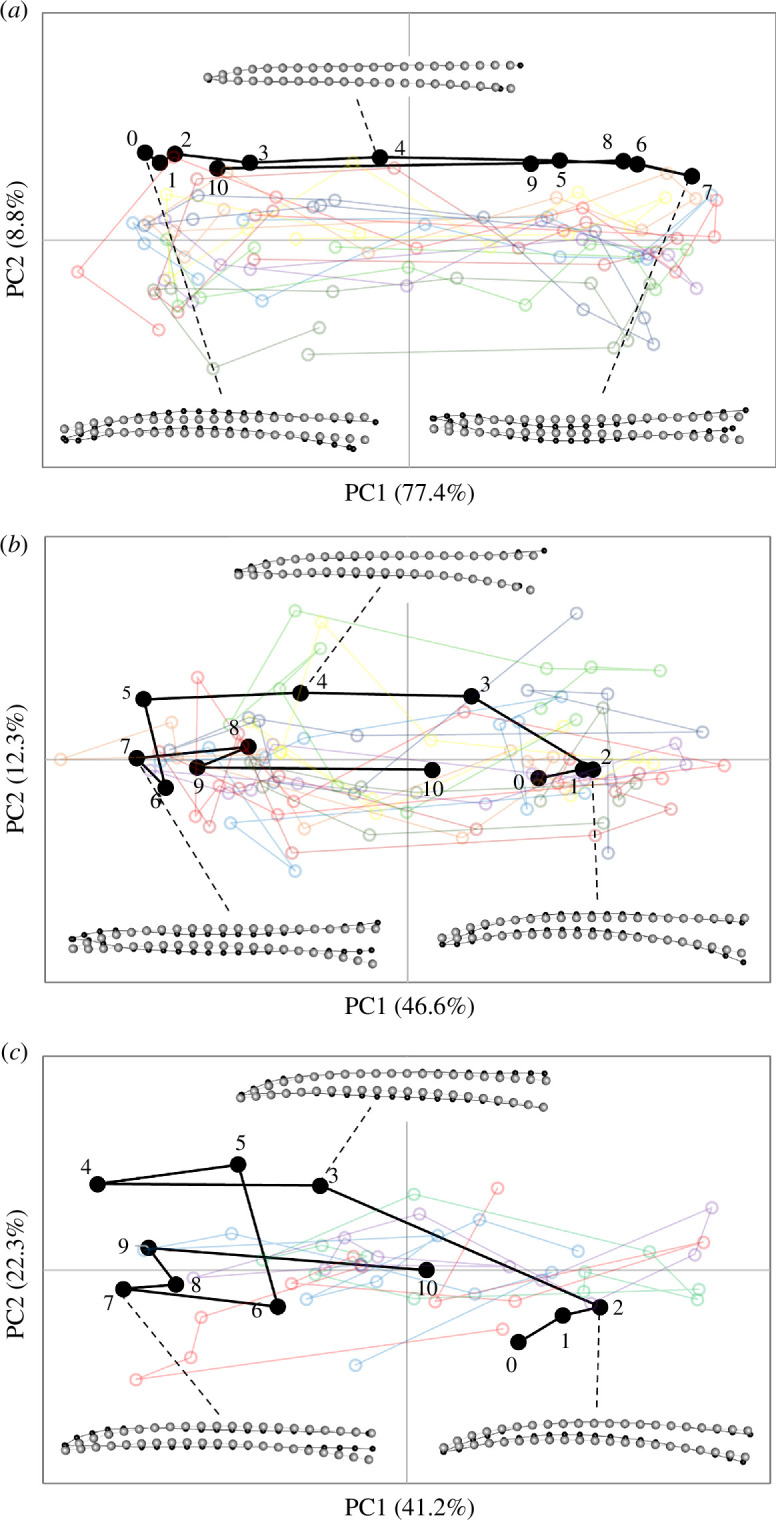
Shape changes in PC1–PC2 morphospace observed in the culmen of species with the most similar, relatively straight, overall bill shapes for (*a*) *Calypte anna*, (*b*) *Amazilis amazilia* and (*c*) *Chalybura buffonii*, during lick cycles. In each graph, ‘lick01’ (black filled symbols, numbered sequentially across frames) is shown for reference; the semi-transparent open symbols and traces in other colours are those of the other nine lick cycles (lick02–10). The insets show representative shape changes (black dots and lines; relative to the grey dots and lines of the consensus configuration, magnified by 7×) for selected frames during lick 01 for each species.

**Figure 4.**
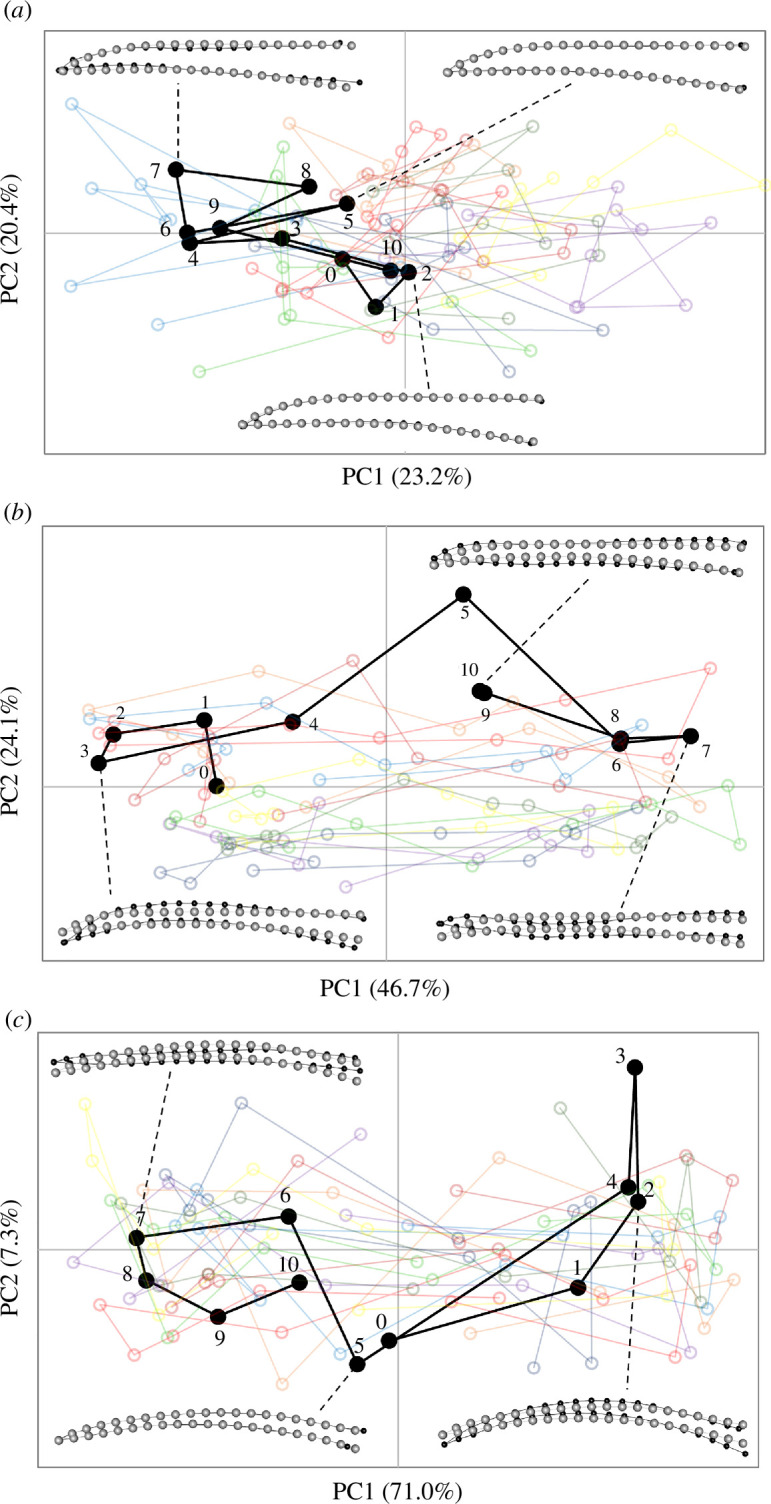
Shape changes in PC1–PC2 morphospace observed in the culmen of species with more aberrant overall bill shapes for (*a*) *Florisuga mellivora*, (*b*) *Myrmia micrura* and (*c*) *Phaethornis longirostris*, during lick cycles. In each graph, ‘lick01’ (black filled symbols, numbered sequentially across frames) is shown for reference; the semi-transparent open symbols and traces in other colours are those of the other nine lick cycles (lick02–10). The insets show representative shape changes (black dots and lines; relative to the grey dots and lines of the consensus configuration, magnified by 7×) for selected frames during lick 01 for each species.

The shapes of the culmens changed during licking as evidenced by their trajectories through morphospace across sequential images (figures 3 and 4; electronic supplementary material, figure S3). The percentages of variance explained by the first two PC axes varied considerably among subjects/taxa, but in each subject the changes in bill shape followed similar trajectories across replicate lick cycles. The Procrustes ANOVAs for each subject revealed that, although the variation among lick cycles was significant, the cycle accounted for a relatively small proportion of the variance (*R^2^* = 0.08−0.25) compared with the significant shape changes detected across frames (*R^2^* = 0.20−0.77; electronic supplementary material, tables S2–S7). However, the degree of deformation in the culmen varied considerably among subjects/taxa, largely owing to differences in overall bill shapes (electronic supplementary material, table S8; figure S10). In general, the culmen exhibited the greatest deformation along the rostral third of the bill, secondarily along the middle third and to varying extents at the base (electronic supplementary material, figure S3). Principal component 1 consistently expressed some degree of relative dorsoventral narrowing versus widening of the culmen, along with a relative straightening versus a relative decurvature of the rostral half of the culmen (figures 3 and 4), from one direction of the PC axis to the other (note, however, that the directions were determined arbitrarily for each separate analysis). Whereas PC1 (which explained the bulk of the variance of each dataset) captured most of the ‘bill bending’ deformation among sequential frames, changes along PC2 were more subtle and typically reflected comparatively minor differences in shape among licking cycles (electronic supplementary material, figures S4–S9).

### 3.2. Flexural rigidity and rhynchokinetic bill movements

Dorsoventral flexural rigidity patterns through the laterally positioned maxillary processes of the premaxillae often differed from patterns observed through the dorsally positioned frontal processes of the premaxillae. We identified three regions with distinct flexural rigidity profiles (electronic supplementary material, figure S11): region 1, which is near the bill base and includes the nasal cavity; region 2, which constitutes most of the bill length and region 3, where the lateral (ventral *sensu* Zusi [17]) and dorsal bars fuse; these regions roughly correspond to the nasal, intermediate and symphysial parts of the prepalatal upper jaw (see fig. 5 in [17]). Within region 2 (the ‘bending zone’ identified from high-speed videography), both the lateral and dorsal bars of the upper bill exhibit substantially lower flexural rigidity values than they generally do in the proximal and distal rigid zones (regions 1 and 3). When the flexural trends through the upper jaw are viewed holistically, the bending zone exhibits substantially lower average dorsoventral flexural rigidity values than either the proximal or distal rigid zones, and this is consistent among species (electronic supplementary material, figures S11 and S12).

**Figure 5.**
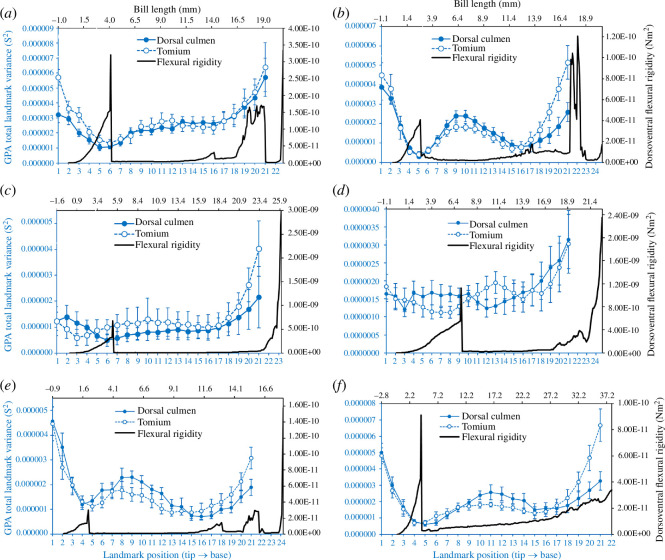
Mean ± 95% CI GPA total landmark variances (S^2^) computed over 11 sequential frames, across *n* = 10 lick cycles for each subject (blue symbols and lines along corresponding x (bottom) and y (left) axes): (*a*) *Amazilis amazilia*, (*b*) *Calypte anna*, (*c*) *Chalybura buffonii*, (*d*) *Florisuga mellivora*, (*e*) *Myrmia micrura* and (*f*) *Phaethornis longirostris*. The blue filled symbols and solid lines correspond to the dorsal culmen and the open symbols and dashed lines correspond to the tomium. The dorsoventral flexural rigidity traces (black, along corresponding x (top) and y (right) axes) of representative specimens of each species from BendCT are superimposed to compare zones of relative structural rigidity with zones of relative kinesis along the lengths of the bills, from the rostral tip to the cranial base (peaks in flexural rigidity correspond to the point of fusion between the premaxillae rostrally and the craniofacial hinge (cranially)). The top horizontal (flexural rigidity bill length) axis starts at negative values accounting for the keratinous rostral tip of the culmen (from the CT scans, see §2); similarly, the bottom horizontal (bill landmark) axis extends beyond 21 as it corresponds to the bony nasofrontal hinge.

These patterns of deformation and landmark displacement during the licking cycle seem to reflect two different forms of motion. On the one hand, the high GPA total variances reflect greater ranges (and speeds) of relative displacements of end members along the vertical (y) axis (orthogonal to the length of the bill), as observed at the distal bill tip. On the other hand, the pattern of variance at the base, and to some extent the mid-region of the bill, are more likely to be a consequence of out-of-plane, lateral expansion and contraction of the bill, drawing the culmen and tomium dorsoventrally in those regions. Thus, in these regions, the observed variance and deformations are indicative of a dorsoventral bending zone (figure 5), probably facilitated by the flattening of the cross-sectional arch of the culmen, which would otherwise resist dorsoventral bending. In most taxa, there is a clear peak in dorsoventral flexural rigidity on either side of which much of the upper bill movement (i.e. ‘bending’) appears to be concentrated (figure 5). Naturally, this varies among taxa; for example, in *Florisuga mellivora* (figure 5*d*), the upper bill movement is diffuse and does not distinctly correspond to the point of minimum dorsoventral flexural rigidity. In other taxa, such as *Amazilis amazilia* (figure 5*a*), *Calypte anna* (figure 5*b*) and *Phaethornis longirostris* (figure 5*f*), there appears to be an additional, more cranially positioned local minimum in dorsoventral flexural rigidity, resulting in an additional bending zone corresponding to high degrees of middle and cranial upper bill motion.

### 3.3. Fluid analysis

**Figure 6.**
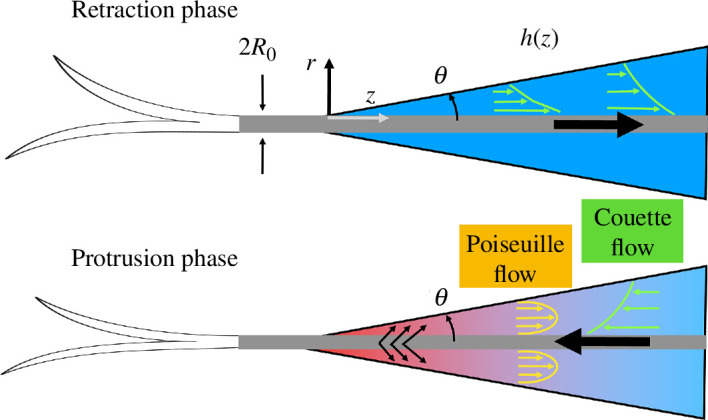
Two schematics showing fluid flows within the bill. (Top) An illustration of the retraction phase, where the tongue is pulled back into the bill, creating a pure shear-driven fluid motion known as Couette flow. (Bottom) An illustration of the protrusion phase; while the bill wrings the tongue, nectar is squeezed into the oral cavity near the tip while the tongue moves outward. The compression at the bill tip generates a high-pressure zone (indicated in red) which diminishes progressively along the tongue’s axis, transitioning to a low-pressure area (shown in blue). This axial pressure gradient gives rise to a pressure-driven flow (Poiseuille flow) that operates concurrently with the shear-driven Couette flow. The combined fluid mechanics during both phases facilitate the efficient transport of nectar.

There are two distinct motions involved in hummingbird drinking: an angle change of the bill walls in relation to the tongue and reciprocating tongue motion. First, a change in the angle of the bill will affect the cross-sectional area of the oral cavity. When the separation between the bill tips is large and the separation at the bill base becomes small, the overall bill angle becomes smaller during the protrusion phase. Inversely, the bill angle increases during the retraction phase. Such an angle change could modify the pressure gradient along the long axis of the bill. When the angle becomes smaller, the pressure on the bill tip will gradually increase, thereby pushing fluid towards the bill base (figure 6). Thus, the angle change between the bill walls and the tongue can contribute to the amount and duration of drinking.

Second, repeated movement of the tongue in and out of the bill can generate a shear flow within the bill, to transport nectar along the bill’s long axis (figure 6). However, the cyclic motion of the tongue by itself would not create a unidirectional flow. To break the symmetry, the bill motion should be coordinated. The squeezing of the tongue by the bill tips wrings the nectar out of the tongue grooves. This motion also results in increasing the bill angle as well as inducing a nectar source inside the oral cavity (figure 6).

To describe fluid motion inside the bill, let $v_{z}\left(r,z\right)$ denote the axial velocity of the fluid within the bill at a radial position r and along the axial position z. The velocity profile inside the bill can be described by


(3.1) $$v_{z}\left(r,z\right)=\frac{1}{4\mu }\frac{dp}{dz}\left[r^{2}-h{\left(z\right)}^{2}\right]\;+\left(U-\frac{1}{4\mu }\frac{dp}{dz}\left[R_{0}^{2}-h{\left(z\right)}^{2}\right]\right)\left(\frac{\ln\left(r/h\left(z\right)\right)}{\ln\left(R_{0}/h\left(z\right)\right)}\right),$$

where μ is the dynamic viscosity of the fluid, dp/dz is the pressure gradient along the axial direction, h(z) is the radius of the fluid column at position z, $R_{0}$ is the radius of the tongue and U is the velocity of the tongue’s linear motion.

The terms with dp/dz represent the flow contribution from the axial pressure gradient and the term with U represents the shear flow due to the tongue’s linear motion. The volumetric flow rate $Q_{0}$ can then be obtained by integrating the axial velocity over the radial cross section, from the tongue ${r=R}_{0}$ to the boundary r=h(z), giving


(3.2) $$Q_{0}\left(z\right)=2\pi \int_{R_{0}}^{h\left(z\right)=\theta z}v_{z}\;rdr.$$

This integral will provide the total flux for a given axial position *z*, assuming axial symmetry of the flow and constant dp/dz


(3.3) $$\begin{array}{l} \;Q_{0}(z)=-\frac{\pi }{8\mu }\frac{dp}{dz}[R_{0}^{2}-h(z{)}^{2}{]}^{2}\\ \;\;+\frac{\pi }{2}\left(U-\frac{1}{4\mu }\frac{dp}{dz}[R_{0}^{2}-h(z{)}^{2}]\right)\left(-2R_{0}^{2}+\frac{R_{0}^{2}-h(z{)}^{2}}{\ln(R_{0}/h(z))}\right)\\ \;\;-\frac{\pi }{8\mu }\;\frac{dp}{dz}=\frac{Q_{0}+\frac{\pi }{2}U\left(2R_{0}^{2}-\frac{R_{0}^{2}-h(z{)}^{2}}{\ln(R_{0}/h(z))}\right)}{(R_{0}^{2}-h(z{)}^{2})\left(\left(R_{0}^{2}-h(z{)}^{2})-(2R_{0}^{2}-\frac{R_{0}^{2}-h(z{)}^{2}}{\ln(R_{0}/h(z))}\right)\right)} \end{array}.$$

The volumetric flow rate Q within the bill must remain invariant with respect to the axial position *z*, satisfying the conservation of mass in the system. Therefore, regardless of the position along the axis of the bill, we assert that $Q=Q_{0}$, where $Q_{0}$ is the volumetric flow rate at a reference cross section.

To estimate the energy cost associated with lapping, we evaluate the wall shear stress on the tongue. This viscous-resisting force is computed by the following relationship:


(3.4) $$\begin{array}{ll} \frac{F}{2\pi R_{0}L}=\mu \frac{\partial v_{z}}{\partial r}{|}_{r=R_{0}} & =\frac{R_{0}}{2}\frac{dp}{dz}+(\mu U-\frac{1}{4}\frac{dp}{dz}[R_{0}^{2}-h(z{)}^{2}])\frac{1}{R_{0}}\\ \;\;\;\;\;\;\;\;\;\;\;\; & =\frac{\mu U}{R_{0}}+\frac{dp}{dz}\left(\frac{R_{0}}{2}-\frac{1}{4R_{0}}\left(R_{0}^{2}-h{\left(z\right)}^{2}\right)\right) \end{array},$$

where τ is the wall shear stress and L is the tongue length inside the bill.


(3.5) $$\frac{\partial v_{z}}{\partial r}=\frac{1}{2\mu }\frac{dp}{dz}r+\left(U-\frac{1}{4\mu }\frac{dp}{dz}(R_{0}^{2}-h(z{)}^{2})\right)\frac{1}{r\ln(R_{0}/h(z))},$$

(3.6) $$\frac{\partial v_{z}}{\partial r}{|}_{r=R_{0}}=\frac{U}{R_{0}\ln(R_{0}/h(z))}-\frac{1}{2\mu }\frac{dp}{dz}\left(\frac{1}{2}(R_{0}^{2}-h(z{)}^{2})\frac{1}{R_{0}\ln(\frac{R_{0}}{h(z)})}-R_{0}\right),$$

$$=\frac{U}{R_{0}\ln(R_{0}/h(z))}-\frac{4}{\pi }\left(\frac{Q_{0}+\frac{\pi }{2}U(2R_{0}^{2}-\frac{R_{0}^{2}-h(z{)}^{2}}{\ln(R_{0}/h(z))}\;)}{\left(R_{0}^{2}-h(z{)}^{2})((R_{0}^{2}-h(z{)}^{2})-(2R_{0}^{2}-\frac{R_{0}^{2}-h(z{)}^{2}}{\ln(R_{0}/h(z))})\right)}\right)\left(\frac{1}{2}(R_{0}^{2}-h(z{)}^{2})\frac{1}{R_{0}\ln(R_{0}/h(z))}-R_{0}\right).$$

In the asymptotic limit $R_{o}\ll h(z)$, the shear stress on the tongue can be approximated. The force per unit length due to shear stress simplifies to


(3.7) $$\frac{F}{2\pi R_{0}L}\approx 2\mu \left(\frac{U}{R_{0}\ln(\frac{R_{0}}{h(z)})}+\frac{Q_{0}}{\pi R_{0}h^{2}}\right)$$

and


(3.8) $$F\approx 4\pi L\;\mu \left(\frac{U}{\ln(\frac{R_{0}}{h(z)})}+\frac{Q_{0}}{\pi h^{2}}\right).$$

Here, the flow rate $Q_{0}$ can be approximated as $\pi R_{0}^{2}U$, which is based on the assumption that hummingbirds efficiently scrape out all nectar from tongue grooves during extraction. Hence, the second term depends on $R_{0}^{2}/h^{2}$, which is slowly varying compared with the logarithmic first term $\ln\left(R_{0}/h\right)$. This implies that the viscous resistance faced by the tongue is significantly affected by the first shear-flow term, particularly by the velocity *U* and bill geometry.


(3.9) $$F\approx 4\pi L\;\mu \frac{U}{\ln(\frac{R_{0}}{h(z)})}.$$

This indicates that increasing the gap between the tongue and the beak, effectively increasing h(z) relative to $R_{0}$, reduces the resisting force due to shear stress. If the bill has a blunt-tip configuration, characterized by a rapid expansion in the height h of the fluid column, the frictional force experienced by the tongue can be substantially reduced. Such a reduction in resisting force is energetically favourable as it potentially lowers the energy required for nectar feeding.

## 4. Discussion

### 4.1. Rhynchokinesis in hummingbirds

Hummingbird maxillae are inherently flexible because both the dorsally positioned bar (bone along the top of the bill, corresponding to the fused frontal processes of the premaxillae) and ventrolaterally positioned bars (bones on the sides of the upper bill, corresponding to the maxillary processes of the premaxillae) are thin, elongated and flattened [17,45]. Nitzsch [50] proposed a form of rhynchokinesis in hummingbirds as an adaptation for nectar feeding, consisting of extensive flexion zones along the dorsal and ventral aspects of the upper jaw (see [14]). However, Zusi [16] found no osteological evidence to support any distal rhynchokinetic capacity in hummingbirds, leaving the prospect of rhynchokinesis in hummingbirds questionable. However, videos presented by Rico-Guevara & Rubega [48] led Zusi [17] to conclude later that distal rhynchokinesis could occur in hummingbirds, possibly through sliding of the ventrolateral bone bars relative to the dorsal bone bar, and intrinsic bending of the ventrolateral bars.

Although the question of hummingbird maxillary bending has not been directly broached in the years since, Rico-Guevara *et al*. [49] showed phase-shifted bill opening where the jaws near the bill tip and the bill base can be separated at different points throughout the licking cycle while maintaining the middle portion of the bill mostly shut during the licking cycle. Such a mechanism could work via bending or rotation of the mandible, ultimately lowering the tip of the lower jaw, by bending the upper jaw to deflect the tip upwards or by combinations of those mechanisms. Given their extensive mandibular flexibility [15,27,28], we expected to find support for mandibular bending to some extent. Instead, our results illustrate clear evidence of upper bill bending in hummingbirds—the first time that maxillary bending near the bill tip has been quantitatively described in this group. Following Zusi’s [16] classification of bill bending, hummingbirds would perform either amphikinesis or distal to extensive rhynchokinesis (for those species previously reported to be prokinetic [17]), or double rhynchokinesis (for those species previously reported to be proximally rhynchokinetic [17]). However, the exact functional categories to which particular hummingbird taxa are best assigned may vary, and clarification must await further anatomical and morphofunctional studies of a wider range of hummingbird species.

By combining high-speed video of bill bending, coupled with a microstructural analysis of bone rigidity, we found that while the mandible (lower jaw) remains relatively in place throughout the licking cycle during nectar feeding, the distal portion—and, to some extent, the middle portion—of the maxilla (upper jaw) bends upwards. With the possible exception of *F. mellivora*, all taxa studied showed discernible rostral, and in some cases middle and cranial, bending zones (where most of the upper bill movement was concentrated) that are punctuated by points of substantially greater dorsoventral flexural rigidity. We loosely interpret these points as ‘hinges’, beyond which areas of much lower dorsoventral flexural rigidity enable the culmen to bend.

The data presented here verify the presence of a region of relatively low dorsoventral flexural rigidity in the upper jaws of several hummingbird taxa. As predicted by high-speed videography, these regions of low flexural rigidity roughly correspond in their position to the region of dorsally directed upper jaw bending and affirm the existence of proximal and distal rigid zones. The dorsal bending of the hummingbird’s upper jaw adds to the simultaneous ventral and lateral deflection of the lower jaws, measured in *Archilochus colubris* during insect hawking events [15], as another means by which hummingbirds achieve enhanced gape sizes. Future work will seek to characterize the mechanism by which this lateral and ventral mandibular deflection occurs; it seems possible that regions of decreased flexural rigidity underlie the ability of hummingbird lower jaws to deflect in this manner as well.

### 4.2. Implications of bill bending for intra-oral fluid transport

To maximize nectar uptake efficiency, offloading as much nectar as possible from the tongue is paramount for maintaining the maximum loading capacity across consecutive licks [45]. After wringing the tongue to unload the nectar inside the bill, this volume of liquid must be transported towards the throat to be swallowed. In hummingbirds, such intra-oral transport must be coordinated with tongue squeezing at the bill tip at rates exceeding several times per second. A bending point in the middle-to-distal region of the bill tip has the potential to coordinate these two processes, by allowing the tip to open and close (to provide nectar on- and off-loading) while keeping the middle-bill region tightly closed to provide a fairly constant internal diameter for the ‘tongue squeegee’ to work properly, wherein the flaps at the base of the tongue can maintain close contact with the intra-oral cavity walls to move the nectar proximally.

Previously, Rico-Guevara *et al*. [49] described intra-oral transport mechanisms during nectar drinking in hummingbirds (distal wringing, tongue raking and basal expansion). Here, we present the functional basis of the maxillary flexibility that allows those mechanisms to occur and be synchronized at high rates. Hummingbirds drink nectar by reciprocating their tongues up to 17 times per second [72]. Bill and tongue movements must, therefore, be well coordinated at this fast pace, as the bill tips must close immediately after the tongue ends its retraction and before it starts its protraction to maximize the amount of nectar retained inside the bill (distal wringing *sensu* Rico-Guevara *et al*. [49]). Nearly closing the bill tips while extruding the tongue through them creates a diminished volume of the distal oral cavity (reduced cross-sectional area) and a sharp angle of the internal bill walls as they contact the tongue (fig. 1 in [45]). This operation is vital not only to move nectar from the tongue to the bill but also to clean and compress the tongue in order to collect as much nectar as possible in the following lick [46].

Distal rhynchokinesis allows for the bill tips to be open during the tongue retraction phase and to be kept mostly closed during the protrusion phase while preventing jaw separation around the middle portion of the bill, which ultimately facilitates the tongue base raking the nectar [49]. A hummingbird’s tongue base is analogous to a stick squeegee used to clean paintball gun barrels, which consists of a rod with a rubber disc at one end that swivels in such a way that when inserted it does not displace the fluid, and when retracted forms a seal against the walls of the barrel pulling fluid in the same direction. The equivalent to the swivelling rubber disc component in hummingbirds is the tongue base with folding flaps (i.e. tongue wings).

Rhynchokinesis near the bill tip allows for close contact between the tongue base flaps and the walls of the intra-oral cavity along the middle portion of the bill while allowing the next aliquot of nectar to be drawn into the bill cavity by the loaded tongue through the open tips. Thus, distal rhynchokinesis enables expansion of the intra-oral space near the bill base to occur in a phase-shifted manner, in such a way that, at the same time that nectar is released into the oral cavity by the nearly closed bill tips, the bill base is open, increasing intra-oral capacity for nectar to flow into an enlarged space towards the throat. We propose that this type of rhynchokinesis near the bill tip improves intra-oral nectar transport and, therefore, feeding efficiency. There is variation across species in the degree of bill bending (e.g. electronic supplementary material, figure S13), which warrants further studies on the potential relationships with their ecology and other selective pressures on bill performance (discussed in §4.3 below). For instance, hummingbirds with short and straight, stout bills might not benefit as much from the advantages of the coordination of intra-oral transport mechanisms described below, but on the other hand, might not need to, given the comparatively limited transport distances for nectar offloading and transport to the throat.

### 4.3. Potential trade-offs and constraints on the evolution of bill bending in hummingbirds

The hummingbird bill can work as a tight unit with clockwork precision coordinating with rapid tongue movements many times a second [31,49]. However, it can also be decoupled as when the gape is expanded by mandibular bowing during displays or arthropod hunting [15,27,28].

Although hummingbirds exhibit the capacity to extensively bend their lower jaws during aerial insectivory [15,27], while drinking nectar hummingbirds hold their mandibles relatively still (e.g. electronic supplementary material, video S1) and appear to use them as a base of support upon which to bend their maxillae while drinking nectar. Since flycatching bill manoeuvres rely on rapid closure by elastic recoil of the mandibular bending [27], it is possible that maxillary bending and passive rapid recoil could also aid during arthropod foraging.

Hummingbird bills are also a key structure to access nectar resources beyond their interaction with flowers. Since a given flower could be depleted by many individuals and often from several species competing in the same area, hummingbirds face interference competition, using their bills as weapons [31,73]. Hence, a bill adaptation expected to enhance fighting proficiency is increased structural soundness to withstand impacts. This would select for overall bill robustness, as more solidly built structures better withstand potentially damaging bending forces and would also transfer larger stabbing and biting forces to the bill tip. Therefore, the maxillary bending and structural flexibility described here could face a trade-off between improving nectar drinking by allowing for opening of the bill tips while the middle portion of the beak remains tightly closed (enhancing intra-oral transport) and improving fighting performance when the bill is used to fend off rivals during territorial quarrels for floral resources. Under the axial load likely to be exerted when the bill is employed as a stabbing weapon, such structural flexibility is likely to cause buckling and even failure. Thus, increased flexural rigidity will improve stabbing performance by diminishing bill bending, but at the same time, it will negatively affect nectar-drinking efficiency.

We expect that the patterns of flexural rigidity (and therefore bending) of hummingbird bills vary intra- and inter-specifically. Adult males are more frequently reported defending floral patches than females and juveniles [74–77], consequently, we expect the bills of males (or individuals that engage in fights more often) to present higher dorsoventral flexural rigidity when compared with females and juveniles. As shown here, there is great variation in bill length (electronic supplementary material, table S10) and size-controlled shape (electronic supplementary material, figure S10) across hummingbird species (with even more variation across the family, e.g. [78]). Flexural rigidity will be affected by such variation in bill architecture, in addition to potential changes in material properties occurring in response to particular selective pressures such as the use of bills as intrasexually selected weapons [79], and as tools for visual displays (e.g. differences in melanization across the bill length, fig. 3 in [49]). For example, a bill trait that greatly affects structural properties is curvature because, when loaded axially, elongated structures are mechanically more resistant to buckling if they are straight [80,81]. As such, bending is disadvantageous for a stabbing weapon, as less force is applied at the tip and subsequently less damage can be done to an opponent. In hummingbirds, straighter bills transmit more force without bending, and pointier bills transform that force into perforation capacity [73]. Therefore, we expect differences arising from pressures of their bills used as weapons, as well as intrinsic differences in bending given their bill architecture (e.g. maxillary thickness and curvature, electronic supplementary material, figure S10).

The extent to which hummingbirds can perform rhynchokinesis near the bill tip depends on the overall shape and size of the bill. We found that the degree of bill bending is greater in bills with relatively more curved distal, and narrower proximal, shapes for their size, and lower in bills characterized by relatively straighter distal, and deeper proximal, shapes for their size (electronic supplementary material, S1.2; figure S13). However, a more comprehensive assessment, including a greater diversity of species in a phylogenetic comparative context (and taking advantage of recent three-dimensional characterization tools, e.g. [78]), is certainly warranted to substantiate this trend.

## 5. Conclusions

We argue that maxillary bending in hummingbirds, arguably the most specialized avian nectarivores, is a functional innovation that improves feeding efficiency and may constitute an important aspect of morphological diversification within plant–hummingbird coevolutionary systems. Hummingbirds can open their bill tips and separate the base of the jaws while maintaining a tightly closed middle section of the bill, which enhances the efficiency of nectar extraction because it allows nectar to be taken up at the bill tips (when offloaded from the tongue) and intraorally transported to the throat (through a tight bill tube) simultaneously. This enables hummingbirds to exploit longer and narrower flowers than they would be able to if their bills were inflexible and if they had to open the entire bill to separate their bill tips. The particularly elongated and narrow corollas that can evolve under this scenario, wherein bills and flower shapes match, would benefit both birds and plants [82], perhaps evolutionarily filtering less efficient pollinators.

## Data Availability

Data and code are available from Zenodo [83]. Supplementary material is available online [84].

## Dedication

Dedicated to the memory of functional morphologist and hummingbird anatomy authority, Richard L. Zusi.

## References

[1] Dumont ER. 2010 Bone density and the lightweight skeletons of birds. Proc. R. Soc. B **277**, 2193–2198. (10.1098/rspb.2010.0117) PMC2880151 20236981

[2] Field DJ, Campbell‐Malone R, Goldbogen JA, Shadwick RE. 2010 Quantitative computed tomography of humpback whale (Megaptera novaeangliae) mandibles: mechanical implications for rorqual lunge‐feeding. Anat. Rec. **293**, 1240–1247. (10.1002/ar.21165) 20583267

[3] Gussekloo SWS, Bout RG. 2005 Cranial kinesis in palaeognathous birds. J. Exp. Biol. **208**, 3409–3419. (10.1242/jeb.01768) 16109900

[4] Field DJ, Lin SC, Ben‐Zvi M, Goldbogen JA, Shadwick RE. 2011 Convergent evolution driven by similar feeding mechanics in balaenopterid whales and pelicans. Anat. Rec. **294**, 1273–1282. (10.1002/ar.21406) 21618438

[5] Rico-Guevara A, Sustaita D, Gussekloo S, Olsen A, Bright J, Corbin C, Dudley R. 2019 Feeding in birds: thriving in terrestrial, aquatic, and aerial niches. In Feeding in vertebrates (eds V Bels, IQ Whishaw), pp. 643–693. Cham, Switzerland: Springer International Publishing. (10.1007/978-3-030-13739-7_17)

[6] Bhullar BAS, Hanson M, Fabbri M, Pritchard A, Bever GS, Hoffman E. 2016 How to make a bird skull: major transitions in the evolution of the avian cranium, paedomorphosis, and the beak as a surrogate hand. Integr. Comp. Biol. **56**, 389–403. (10.1093/icb/icw069) 27371392

[7] Benito J, Kuo PC, Widrig KE, Jagt JWM, Field DJ. 2022 Cretaceous ornithurine supports a neognathous crown bird ancestor. Nature **612**, 100–105. (10.1038/s41586-022-05445-y) 36450906

[8] Simonetta AM. 1960 On the mechanical implications of the avian skull and their bearing on the evolution and classification of birds. Q. Rev. Biol. **35**, 206–220. (10.1086/403106)

[9] Bock WJ. 1964 Kinetics of the avian skull. J. Morphol. **114**, 1–41. (10.1002/jmor.1051140102)

[10] Bock WJ. 1999 Avian cranial kinesis revisited. Acta Ornithol. **34**, 115–122. https://www.researchgate.net/publication/281317797_Cranial_kinesis_revisited

[11] Zweers GA, Vanden Berge JC, Berkhoudt H. 1997 Evolutionary patterns of avian trophic diversification. Zool. Jena. **100**, 25–27. https://www.researchgate.net/publication/246122841_Evolutionary_pattern_of_avian_trophic_diversification

[12] Bout RG, Zweers GA. 2001 The role of cranial kinesis in birds. Comp. Biochem. Physiol. A Mol. Integr. Physiol. **131**, 197–205. (10.1016/S1095-6433(01)00470-6) 11733177

[13] Holliday CM, Witmer LM. 2008 Cranial kinesis in dinosaurs: intracranial joints, protractor muscles, and their significance for cranial evolution and function in diapsids. J. Vertebr. Paleontol. **28**, 1073–1088. (10.1671/0272-4634-28.4.1073)

[14] Bühler P. 1981 Functional anatomy of the avian jaw apparatus. In Form and function in birds (eds AS King, J McLelland), pp. 439–468. London, UK: Academic Press.

[15] Yanega GM, Rubega MA. 2004 Hummingbird jaw bends to aid insect capture. Nature **428**, 615–615. (10.1038/428615a) 15071586

[16] Zusi RL. 1984 A functional and evolutionary analysis of rhynchokinesis in birds. Washington, DC: Smithsonian Institution Press. (10.5479/si.00810282.395)

[17] Zusi RL. 2013 Introduction to the skeleton of hummingbirds (Aves: Apodiformes, Trochilidae) in functional and phylogenetic contexts. Ornithol. Monogr. **77**, 1–94. (10.1525/om.2013.77.1.1)

[18] Zweers GA, Gerritsen AFC. Transitions from pecking to probing mechanisms in waders. Neth. J. Zool. **47**, 161–208. (10.1163/156854297X00166)

[19] Gussekloo SWS, Vosselman MG, Bout RG. 2001 Three-dimensional kinematics of skeletal elements in avian prokinetic and rhynchokinetic skulls determined by Roentgen stereophotogrammetry. J. Exp. Biol. **204**, 1735–1744. (10.1242/jeb.204.10.1735) 11316494

[20] Rubega MA, Obst BS. 1993 Surface-tension feeding in phalaropes: discovery of a novel feeding mechanism **110**, 169–178. https://sora.unm.edu/node/25233

[21] Prakash M, Quéré D, Bush JWM. 2008 Surface tension transport of prey by feeding shorebirds: the capillary ratchet. Science **320**, 931–934. (10.1126/science.1156023) 18487193

[22] Rubega MA. 1997 Surface tension prey transport in shorebirds: how widespread is it? Ibis **139**, 488–493. (10.1111/j.1474-919X.1997.tb04663.x)

[23] Estrella SM, Masero JA. 2007 The use of distal rhynchokinesis by birds feeding in water. J. Exp. Biol. **210**, 3757–3762. (10.1242/jeb.007690) 17951416

[24] Mayr G. 2002 Osteological evidence for paraphyly of the avian order Caprimulgiformes (nightjars and allies). J. Ornithol. **143**, 82–97. (10.1007/BF02465461)

[25] Nesbitt SJ, Ksepka DT, Clarke JA. 2011 Podargiform affinities of the enigmatic fluvioviridavis platyrhamphus and the early diversification of strisores (“Caprimulgiformes” + Apodiformes). PLoS One **6**, e26350. (10.1371/journal.pone.0026350) 22140427 PMC3227577

[26] Chen A, White ND, Benson RBJ, Braun MJ, Field DJ. 2019 Total-evidence framework reveals complex morphological evolution in nightbirds (Strisores). Diversity **11**, 143. (10.3390/d11090143)

[27] Smith ML, Yanega GM, Ruina A. 2011 Elastic instability model of rapid beak closure in hummingbirds. J. Theor. Biol. **282**, 41–51. (10.1016/j.jtbi.2011.05.007) 21609721

[28] Chaparro-Herrera S, Espejo Delgado NR, Ceruche-Cubillos K, Lopera-Salazar A, Rico-Guevara A. 2019 Streptognathism displays in two Phaethornis hermits. Cotinga **41**, 114–117. https://www.researchgate.net/publication/335107167_Streptognathism_displays_in_two_Phaethornis_hermits

[29] Mayr G. 2003 Phylogeny of early tertiary swifts and hummingbirds (Aves: Apodiformes). Auk **120**, 145. (10.1642/0004-8038(2003)120[0145:POETSA]2.0.CO;2)

[30] Mayr G. 2004 Old World fossil record of modern-type hummingbirds. Science **304**, 861–864. (10.1126/science.1096856) 15131303

[31] Rico-Guevara A, Rubega MA, Hurme KJ, Dudley R. 2019 Shifting paradigms in the mechanics of nectar extraction and hummingbird bill morphology. Integr. Org. Biol. **1**, oby006. (10.1093/iob/oby006) 33791513 PMC7671138

[32] Song M, Sukumar M, Raju CSK, Varma SVK, Ijaz Khan M, Awwad FA, Ismail EAA. 2023 Inspection of Couette and pressure-driven Poiseuille entropy-optimized dissipated flow in a suction/injection horizontal channel: analytical solutions. Open Phys. **21**, 20230109. (10.1515/phys-2023-0109)

[33] Walton A, Yu K. 2024 The linear stability of plane Couette flow with a compliant boundary. J. Eng. Math. **144**, 1. (10.1007/s10665-023-10307-1)

[34] Pandey A, Chen ZY, Yuk J, Sun Y, Roh C, Takagi D, Lee S, Jung S. 2023 Optimal free-surface pumping by an undulating carpet. Nat. Commun. **14**, 7735. (10.1038/s41467-023-43059-8) 38007556 PMC10676362

[35] Gart S, Socha JJ, Vlachos PP, Jung S. 2015 Dogs lap using acceleration-driven open pumping. Proc. Natl Acad. Sci. USA **112**, 15798–15802. (10.1073/pnas.1514842112) 26668382 PMC4703018

[36] Reis PM, Jung S, Aristoff JM, Stocker R. 2010 How cats lap: water uptake by Felis catus. Science **330**, 1231–1234. (10.1126/science.1195421) 21071630

[37] Crompton AW, Musinsky C. 2011 How dogs lap: ingestion and intraoral transport in Canis familiaris. Biol. Lett. **7**, 882–884. (10.1098/rsbl.2011.0336) 21613282 PMC3210653

[38] Wei J, Huo Z, Gorb SN, Rico-Guevara A, Wu Z, Wu J. 2020 Sucking or lapping: facultative feeding mechanisms in honeybees (Apis mellifera). Biol. Lett. **16**, 20200449. (10.1098/rsbl.2020.0449) 32780979 PMC7480147

[39] Wei J, Rico-Guevara A, Nicolson SW, Brau F, Damman P, Gorb SN, Wu Z, Wu J. 2023 Honey bees switch mechanisms to drink deep nectar efficiently. Proc. Natl Acad. Sci. USA **120**, e2305436120. (10.1073/pnas.2305436120) 37459520 PMC10372696

[40] Rico-Guevara A, Rubega MA. 2012 Hummingbird feeding mechanics: comments on the capillarity model. Proc. Natl Acad. Sci. USA **109**, E867–E867. (10.1073/pnas.1119750109) 22460801 PMC3326478

[41] Samy RA, Suthanthiraraj PPA, George D, Iqbal R, Sen AK. 2019 Elastocapillarity-based transport of liquids in flexible confinements and over soft substrates. Microfluid. Nanofluidics **23**, 100. (10.1007/s10404-019-2266-2)

[42] Cuban D, Hewes AE, Sargent AJ, Groom DJE, Rico-Guevara A. 2022 On the feeding biomechanics of nectarivorous birds. J. Exp. Biol. **225**, jeb243096. (10.1242/jeb.243096) 35048977

[43] Hewes AE, Baldwin MW, Buttemer WA, Rico-Guevara A. 2023 How do honeyeaters drink nectar? Integr. Comp. Biol. **63**, 48–58. (10.1093/icb/icad048) 37279913

[44] Hewes AE, Cuban D, Groom DJE, Sargent AJ, Beltrán DF, Rico-Guevara A. 2022 Variable evidence for convergence in morphology and function across avian nectarivores. J. Morphol. **283**, 1483–1504. (10.1002/jmor.21513) 36062802

[45] Rico-Guevara A, Rubega MA. 2017 Functional morphology of hummingbird bill tips: their function as tongue wringers. Zoology **123**, 1–10. (10.1016/j.zool.2017.06.001) 28760683

[46] Rico-Guevara A, Fan TH, Rubega MA. 2015 Hummingbird tongues are elastic micropumps. Proc. R. Soc. B **282**, 20151014. (10.1098/rspb.2015.1014) PMC4632618 26290074

[47] Rico-Guevara A. 2014 Morphology and function of the drinking apparatus in hummingbirds. Doctoral dissertation, University of Connecticut, Storrs, CT. See https://opencommons.uconn.edu/dissertations/490.

[48] Rico-Guevara A, Rubega MA. 2011 The hummingbird tongue is a fluid trap, not a capillary tube. Proc. Natl Acad. Sci. USA **108**, 9356–9360. (10.1073/pnas.1016944108) 21536916 PMC3111265

[49] Rico-Guevara A, Hurme KJ, Rubega MA, Cuban D. 2023 Nectar feeding beyond the tongue: hummingbirds drink using phase-shifted bill opening, flexible tongue flaps and wringing at the tips. J. Exp. Biol. **226**, jeb245074. (10.1242/jeb.245074) 37010268 PMC10112966

[50] Nitzsch CL. 1816 Über die bewegung des oberkiefers der vögel. Dtsch. Arch. Physiol. **2**, 361–380. http://vlp.mpiwg-berlin.mpg.de/references?id=lit14031

[51] Moller W. 1930 Über die Schnabel- und Zungenmechanik blütenbesuchender Vögel. I Biol. Gen. **VI**, 651–726. https://scholar.google.com/scholar_lookup?journal=Biol.%20Generalis&title=%C3%9Cber%20die%20Schnabel-%20und%20Zungenmechanik%20bl%C3%BCtenbesuchender%20V%C3%B6gel.%20I&volume=VI&publication_year=1930&pages=651-726&amp;

[52] Moller W. 1931 Bemerkungen zu Scharnke’s Mitteilung 'Die Nektaraufnahme mit der Kolibrizunge' Ornithol. Monatsber. **39**, 135–138. https://scholar.google.com/scholar_lookup?journal=Ornithol.%20Monatsber.&title=Bemerkungen%20zu%20Scharnke%27s%20Mitteilung%20%E2%80%98Die%20Nektaraufnahme%20mit%20der%20Kolibrizunge%E2%80%99&author=W.%20Moller&volume=39&publication_year=1931&pages=135-138&amp;

[53] Moller W. 1932 Die zungen der kostarizensischen zuckervögel. Z. Mikr. Anat. Forsch. **28**, 363–417. https://scholar.google.com/scholar_lookup?journal=Z.%20Mikr.-anat.%20Forsch.&title=Die%20Zungen%20der%20kostarizensischen%20Zuckerv%C3%B6gel&author=W.%20Moller&volume=28&publication_year=1932&pages=363-417&amp;

[54] Ortiz-Crespo F. 1972 A new method to separate immature and adult hummingbirds. Auk **89**, 851–857. (10.2307/4084114)

[55] Yanega GM, Pyle P, Geupel GR. 1997 The timing and reliability of bill corrugations for ageing hummingbirds. West. Birds **28**, 13–18.

[56] Carnes B, Ash A. 2023 Many Central American hummingbirds can be aged and sexed by molt patterns and bill corrugations. J. Field Ornithol. **94**, art11. (10.5751/JFO-00305-940311)

[57] Schneider CA, Rasband WS, Eliceiri KW. 2012 NIH image to imageJ: 25 years of image analysis. Nat. Methods **9**, 671–675. (10.1038/nmeth.2089) 22930834 PMC5554542

[58] Rohlf FJ. 2010 tpsDig, digitize landmarks and outlines. New York, NY: Department of Ecology and Evolution, State University of New York.

[59] Moccetti P *et al*. 2023 Is shape in the eye of the beholder? Assessing landmarking error in geometric morphometric analyses on live fish. PeerJ **11**, e15545. (10.7717/peerj.15545) 37605749 PMC10440062

[60] Adams DC, Cerney MM. 2007 Quantifying biomechanical motion using Procrustes motion analysis. J. Biomech. **40**, 437–444. (10.1016/j.jbiomech.2005.12.004) 16448654

[61] Martinez CM, McGee MD, Borstein SR, Wainwright PC. 2018 Feeding ecology underlies the evolution of cichlid jaw mobility. Evolution **72**, 1645–1655. (10.1111/evo.13518) 29920668

[62] Martinez CM, Wainwright PC. 2019 Extending the geometric approach for studying biomechanical motions. Integr. Comp. Biol. **59**, 684–695. (10.1093/icb/icz104) 31199437

[63] Rohlf FJ. 2019 TPSrelw32: relative warps. New York, NY: NY State University at Stony Brook.

[64] Perez SI, Bernal V, Gonzalez PN. 2006 Differences between sliding semi-landmark methods in geometric morphometrics, with an application to human craniofacial and dental variation. J. Anat. **208**, 769–784. (10.1111/j.1469-7580.2006.00576.x) 16761977 PMC2100233

[65] Adams DC, Collyer ML. 2018 Multivariate phylogenetic comparative methods: evaluations, comparisons, and recommendations. Syst. Biol. **67**, 14–31. (10.1093/sysbio/syx055) 28633306

[66] Adams DC, Collyer ML. 2019 Phylogenetic comparative methods and the evolution of multivariate phenotypes. Annu. Rev. Ecol. Evol. Syst. **50**, 405–425. (10.1146/annurev-ecolsys-110218-024555)

[67] Collyer ML, Adams DC. 2021 RRPP: linear model evaluation with randomized residuals in a permutation procedure. (10.32614/CRAN.package.RRPP)

[68] Adams DC, Otárola‐Castillo E. 2013 geomorph: an R package for the collection and analysis of geometric morphometric shape data. Methods. Ecol. Evol. **4**, 393–399. (10.1111/2041-210X.12035)

[69] Zelditch ML, Swiderski DL, Sheets HD. 2012 Geometric morphometrics for biologists: a primer, 2nd ed. New York, NY: Academic Press.

[70] Campbell-Malone R. 2007 Biomechanics of North Atlantic right whale bone: mandibular fracture as a fatal endpoint for blunt vessel-whale collision modeling. Doctoral thesis in Biological Oceanography, Massachusetts Institute of Technology/Woods Hole Oceanographic Institution, Cambridge, MA.

[71] Ciarelli MJ, Goldstein SA, Kuhn JL, Cody DD, Brown MB. 1991 Evaluation of orthogonal mechanical properties and density of human trabecular bone from the major metaphyseal regions with materials testing and computed tomography. J. Orthop. Res. **9**, 674–682. (10.1002/jor.1100090507) 1870031

[72] Ewald PW, Williams WA. 1982 Function of the bill and tongue in nectar uptake by hummingbirds. Auk. **99**, 573–576. https://www.jstor.org/stable/4085938

[73] Rico-Guevara A, Araya-Salas M. 2015 Bills as daggers? A test for sexually dimorphic weapons in a lekking hummingbird. Behav. Ecol. **26**, 21–29. (10.1093/beheco/aru182)

[74] Temeles EJ, Pan IL, Brennan JL, Horwitt JN. 2000 Evidence for ecological causation of sexual dimorphism in a hummingbird. Science **289**, 441–443. (10.1126/science.289.5478.441) 10903203

[75] Brown JH, Kodric-Brown A. 1979 Convergence, competition, and mimicry in a temperate community of hummingbird‐pollinated flowers. Ecology **60**, 1022–1035. (10.2307/1936870)

[76] Temeles EJ, Goldman RS, Kudla AU. 2005 Foraging and territory economics of sexually dimorphic purple-throated caribs (Eulampis jugularis) on three Heliconia morphs. Auk **122**, 187–204. (10.1093/auk/122.1.187)

[77] Wolf LL. 1975 Female territoriality in the purple-throated carib. Auk **92**, 511–522. (10.2307/4084604)

[78] Medina J *et al*. 2024 PicoCam: high-res. 3D imaging of live animals and preserved specimens. bioRxiv. (10.1101/2024.07.16.603742)

[79] Rico-Guevara A, Hurme KJ. 2019 Intrasexually selected weapons. Biol. Rev. **94**, 60–101. (10.1111/brv.12436) 29924496

[80] Dahlberg T. 2004 Procedure to calculate deflections of curved beams. Int. J. Eng. Educ. **20**, 503–513.

[81] Kuo S, Yang Y. 1991 New theory on buckling of curved beams. J. Eng. Mech. **117**, 1698–1717. (10.1061/(ASCE)0733-9399(1991)117:8(1698))

[82] Rico-Guevara A, Hurme KJ, Elting R, Russell AL. 2021 Bene'fit' assessment in pollination coevolution: mechanistic perspectives on hummingbird bill-flower matching. Integr. Comp. Biol. **61**, 681–695. (10.1093/icb/icab111) 34050734

[83] Rico-Guevara A, Sustaita D, Hurme K, Hanna J, Jung S, Field D. 2024 Data from: Upper bill bending as an adaptation for nectar feeding in hummingbirds. Zenodo. (10.5281/zenodo.13896413) PMC11601124 39601640

[84] Rico-Guevara A, Sustaita D, Hurme KJ, Hanna JE, Jung S, Field D. 2024 Supplementary material from: upper bill bending as an adaptation for nectar feeding in hummingbirds. Figshare. (10.6084/m9.figshare.c.7548191) PMC11601124 39601640

